# Current progress on murals: distribution, conservation and utilization

**DOI:** 10.1186/s40494-023-00904-9

**Published:** 2023-03-27

**Authors:** Yihui Wang, Xiaodong Wu

**Affiliations:** 1 College of Fine Arts and Design, Lanzhou University of Arts and Science, 400 Yanbei Road, Lanzhou, 730000 China; 2 Cryosphere Research Station on the Qinghai-Tibet Plateau, State Key Laboratory of Cryospheric Science, Northwest Institute of Eco-Environment and Resources, Chinese Academy of Sciences, Lanzhou, 730000 China

**Keywords:** Conservation, Chemical composition, Cultural heritage, Grotto, Non-invasive technique, Tourism

## Abstract

As non-renewable cultural heritages, murals have important implications in historical customs, religions, and philosophy as well as their aesthetic values. Recently, many murals are threatened by natural factors and human activities. During the past decades, there are increasing interest in the investigation of murals. Here we review the current status of murals and provide an up-to-date summary of achievements related to murals. The murals that draw the most attention are distributed in Mexico, Ireland, China, and Spain. The aesthetics, history, cultural, educational, and economic values of murals are comprehensively analyzed. The main research technologies used to detect the chemical compositions and physical structures of murals are also summarized. The restoration of murals includes several procedures such as stabilization, repair, surface cleaning, and pigment reconversion. Emerging technologies such as computer science benefit the research and conservation of murals. We also propose that tourism management and climate change should be incorporated into the conservation of murals in the future.

## Introduction

A Mural is a painting on a wall, ceiling, bridge, or other permanent substrates, it is usually connected with architecture. The murals are widely distributed around the world. The earliest murals can date to about 52,000–40,000 BP as cave paintings in *Borneo* [1], and about 32,000 BP in *Chauvet* cave in France [2]. In ancient *Egyptian* tombs which were built around 3150 BP, many ancient murals have been found. These pre-historic murals were used to convey messages or stories of former lifestyles or habits. It has been suggested that communication through paintings on walls eventually formed a writing system [3].

As a visual cultural heritage of historical recording information, murals, especially ancient murals, are precious heritages because they reflect the life and cultural advances of human history as well as enormous value to humanity [4]. They represent a masterpiece of human creative genius, and exhibit an important interchange of human values [5]. Therefore, murals have significant historical, aesthetic, ethnological, anthropological, and scientific values.

**Fig. 1:**
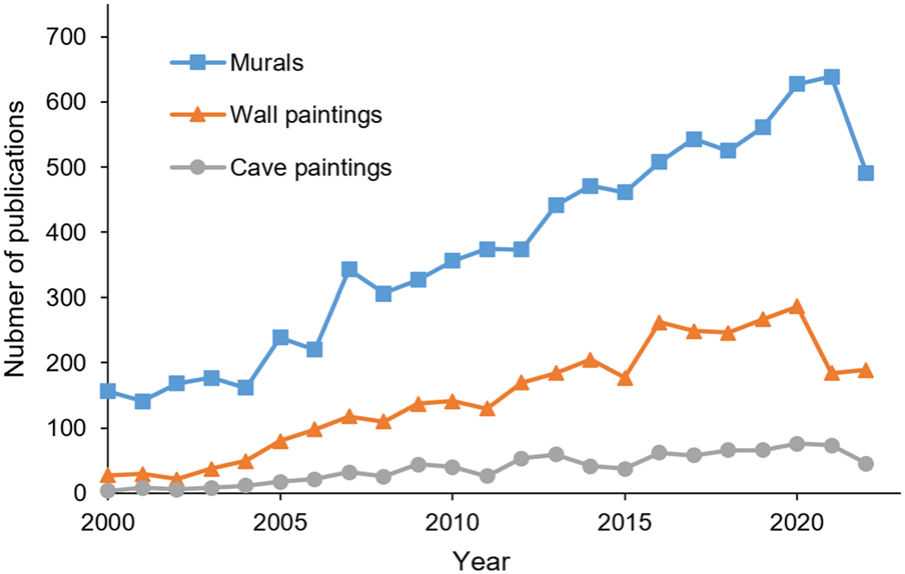
Number of publications in the web of science through time for various search terms associated with murals, wall paintings, and cave paintings. The search was based on the data from the ISI Web of Science databases including Science Citation Index Expanded (SCI-Expanded) and Social Sciences Citation Index (SSCI), and the search was conducted on December 2, 2022

There is increasing attention in the research and conservation of murals during the past decades. To provide context on the relative activity concerned with murals, we performed the search at the end of 2022 using the “All Fields” categorical function, and the baseline period is the last 22 years. Based on the Web of Science (Clarivate Analysis) databases including both Science Citation Index Expanded (SCI-Expanded) and Social Sciences Citation Index (SSCI), the yearly publication counts since 2000 were extracted. It is clear that the results from the words of murals, wall paintings, and cave paintings showed similar increasing trends (Fig. 1). However, due to the natural degradation of the materials of the murals, effects of changing environmental conditions, heightened human activities, increasing pollution, as well as natural hazards including earthquake, rainstorms, floods, and rockfall, many ancient murals were damaged to varying degrees [4]. The damages and deteriorations of murals strongly affect their values and impose great risks to the conservation of this type of cultural heritage [6].

The traditional restoration of murals mainly depends on the manual implementation, these methods are time-consuming and expensive, while the efficiency is usually low. These methods are irreversible and risky, and thereby restrict the development of mural protection [7]. During the past decades, there are emerging new methods for the detection of mural materials composition and multiple-layer structures [8, 9]. Based on the physical, chemical, and biological analysis, the processes and mechanisms of the deterioration have been extensively discussed [10–12]. Meanwhile, new materials and methods have been also developed for the conservation of murals [13–15]. In addition to on-site study and conservation measures, machine learning [6, 16], deep learning [17, 18], and virtual reality methods [19–21] are widely used for the detection and conservation of murals with the rapid development of computer science and technology. These progresses greatly improve our ability to study and conserve murals although the modern restoration of murals is still time-consuming and complicated work.

Many murals are also important resources for tourism and education. Once damaged, the murals are not recoverable and thus the heritages should be protected for sustainable development. The fundamental solution is to protect the intact murals against deterioration under natural and anthropogenic influences. During the past decades, although many achievements have been made to protect the murals [22], many areas face challenges to conserve murals, and it is also challenging to balance modernization and heritage protection [23]. In this review, the progress in the distribution, values, chemical composition, physical structure, deterioration, and its driving mechanisms, restoration, and protection of murals are synthesized. The future directions of mural protection are also described. The objective of this study is to provide an important scientific frame for the future conservation and utilization of murals by synthesizing the findings on murals.

## Major distribution areas of murals

Although there is no strict rule to define the modern and ancient mural arts, murals that were painted since the nineteenth century are usually regarded as modern murals, which are distributed in many cities such as Madrid, New York, London, Paris, Mexico, and some of them become famous tourist destinations [3]. For the ancient murals, the magnitude, conservation status, and values varied greatly. Some ancient murals with high values alone themselves or together with other cultural or natural components have been added to the world heritage sites (https://whc.unesco.org/en/list/), which are designated by United Nations Educational, Scientific and Cultural Organization (UNESCO). Many ancient murals are distributed in imperial palaces, mausoleums, caves, temples, ancient cities, and archaeological remains that are listed in world heritage sites.

**Fig. 2:**
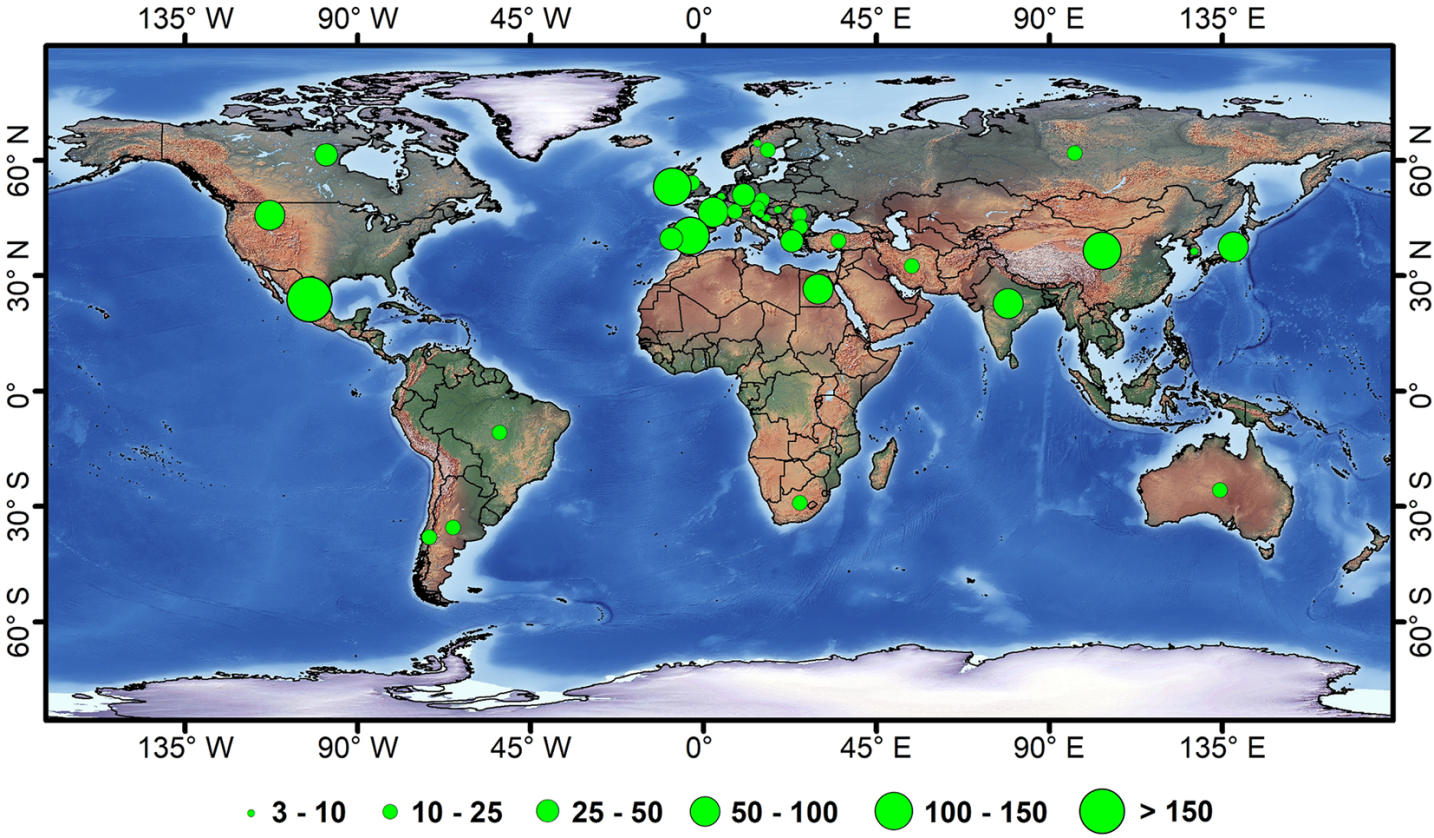
The number of all the publications in the web of science till 2022. The search was based on the data from the ISI Web of Science databases including Science Citation Index Expanded (SCI-Expanded) and Social Sciences Citation Index (SSCI), and the search was conducted on December 2, 2022. The figure was created using ENVI 5.3 (Exelis Inc., Boulder, CO)

There are some regional mural data have been established. The Web Gallery of Art (https://www.wga.hu/index1.html) is a virtual museum and a searchable database that contains some murals in Europe from the 3rd to nineteenth centuries. It serves multiple purposes including a source of artistic enjoyment, a convenient alternative to visit a distant museum, a tool for education, etc. The Rock Art Database (https://rockartdatabase.com/) focuses on the arts that are painted on the rock. This database is a non-profit online project at Griffith University in Australia, and it seeks to improve theory and practice in the digital curation of rock data. The Mural and Street Art Databases (https://guides.canadacollege.edu/murals/databases) provide several links for different books and mural data in different areas. The datasets mainly belong to street art, and most of the murals contained in the databases are modern graffiti. There are no global datasets for murals due to the wide distribution and the enormous number of murals. The publication number in different areas were analyzed. Although the publication number is not identical to the number and importance of the murals since publication on international journals is affected by other factors such as the language, and development of science and technology in these countries, it is definitely a useful indicator of the public’s level of concern for murals. Based on the Web of Science (Clarivate Analysis) databases including both Science Citation Index Expanded (SCI-Expanded) and Social Sciences Citation Index (SSCI), the search on December 2 in 2022 using the “topic” categorical function with the inputting words of “murals” and the name of each country we re-conducted. All the publication counts to show the spatial distribution of publications numbers were collected (Fig. 2). The highest numbers were recorded in Mexico (157), Ireland (135), China (128), and Spain (111), followed by the United States (84), France (63), Japan (52), and Egypt (51). Here the typical murals in Europe, America, and Asia were briefly introduced.

Europe has the highest number of publications in murals. The earlier murals were found in ancient cities or tombs. For example, the ancient city of *Ostia* (Italy), which can date back to the second century had many murals on buildings [24], and the Etruscan tombs that were decorated with murals in Italy were developed during a period from the 7th to the third century BCE [25]. However, the murals largely were destroyed and only can be studied by the fragments in the ancient city [24], and the murals in tombs had suffered from physic-chemical and biological weathering due to the temperature and humid regimes [25]. Currently, the murals that received extensive attention are usually associated with the building in churches, and most of these murals were painted in the Medieval period. The church of *Sotterra* in the province of *Cosenza*, Italy, is probably the *Byzantine* origin. The frescoes in this church, which date back to the 9th to tenth century, present a direct derivation from the *Byzantine* stylistic features [26]. In the rupestrian church *Grotta del Crocifisso* (Lentini, Italy), the earliest mural paintings date back to the twelfth century, and these wall paintings constitute the greatest wealth of this church [27]. Located in *Castellammare del Golfo* (*Trapani,* Italy), *Santa Margherita*’s cave is a natural cave, containing the remains of paintings, which can date back to thetwelfth century. This cave is a karstic cave, located on a coastal cliff. Due to the humid conditions and sea aerosols which influenced the salt composition and microbial community, the murals are now in a poor state of conservation [28]. The *Museo Nacional del Prado* in Spain is composed of several buildings, and some of them were built in the seventeenth century have famous murals [29]. In southern Sweden, all the Romanesque murals are dated to around the thirteenth century [30]. In Russia, the *St. George Cathedral* was built in the first half of the twelfth century. The wall paintings covered all the walls, some stairs towers, and the cupola that crowns the tower. However, due to the fire damage, most of the decoration of the interior was replaced in the first half of the nineteenth century, and the original the twelfth century murals were preserved only in the stairs tower. During 2013–2017, a large number of pieces of knocked-down the twelfth century murals were discovered below the floor level. Some of the large fragments are preserved in high-quality paintings and can be used for mural restoration [31]. In central Europe (Austria, Slovakia, Hungary, Slovenia, Croatia), many churches were established around 1400 because central Europe was an important crossroad of political, economic, social, cultural, and artistic currents at that time. Many parts of these churches were painted with murals. The murals from the fourteenth century show strong Italian *Trecento* influences, while those from the fifteenth century have more local impacts [32]. Northern Ireland has around 2000 murals, which mainly belong to modern morals. These murals can be traced back to 1908 when the Protestants began painting murals to celebrate the victory in the Battle of the *Boyne* in 1690 [33]. The largest concentration of murals was found in *Belfast*, which has at least 700 murals. Many of these murals clearly showed political conflicts [34, 35].

America has a rich mural heritage, and this is especially true of Mexico, Canada, and the United States. These murals show many features of their history, culture, politics, and even their education or advertising [36]. In Mexico, the murals in *Teotihuacan*, which is considered a major cultural and political center of ancient Mesoamerica, were painted embedded since the early stages (1–200 CE) and until the collapse of the great city (650 CE) [37, 38], and these beautiful and complex murals are considered the important source of understanding *Teotihuacan* culture and society [39]. Despite the ancient murals in Mexico can be traced back to Mayan Empire [40, 41], the most famous murals in Mexico were created in the modern era. The major creation of murals in Mexico began in the 1920s when the Mexican government attempted to reunify the country under the government post-Mexican revolution [42]. From the 1920s to the 1970s, many murals with nationalistic, social, and political messages were pained in many public settings, most of them located in historical buildings and museums [43].

In Asia, there are many ancient tombs and buildings that have murals. These murals are painted in different periods that span a wide time period. Some of these murals are famous. For example, the *Goa-ri Tomb* in the *Republic of Korea*, has murals that were painted in the sixth century. Many of the wall murals were missing at the time of their discovery, and the remaining murals have immense value as cultural heritage [44]. Although many other murals in tombs or ancient buildings in Asia may be small and not famous, they are still valuable resources for history researchers [45]. The most important murals found in Asia were discovered in the areas along the Silk Roads, and several famous murals sites are distributed from central Asian countries to China. The *Mogao Grottoes* in northwest China, represent the greatest achievement from the 4th to the fourteenth century. They were first constructed in 366 CE [46]. These grottoes preserve the largest underground galleries in the world, and there are currently more than 2000 painted sculptures in the 487 caves. A total of 45,000 m^2^ of murals in these grottoes show various aspects of historical politics, economics, culture, arts, ethnic relations, and daily dress [47]. The murals in these grottoes accounted for most of the studies in China. Among the 128 publications on the international journal in China (Fig. 2), 102 of them focused on *Mogao Grottoes*. In Northwest China, there is another grotto site that has been inscribed on the World Heritage List, namely, the *Kizil Grottoes.* These grottoes are regarded as the earliest major *Buddhist* cave complex in northwest China. These grottoes were excavated in the third century and ended in the ninth century [48]. There are 236 cave temples in *Kizil* that stretch at a length of 2 km. Although many caves have been damaged and looted, there are about 5000 m^2^ murals remaining, mostly depicting *Jakat* stories, *avadanas*, and legends of the *Buddha* [49]. In addition to the *Buddha* culture, *Jesus Christ* also appears in Asian murals. For example, the *St. Mary’s Orthodox Syrian* Church in southern India, which was built in the sixteenth century, is a treasure trove of mural art. The murals depict the major events in the life of *Jesus Christ*, the *Lord’s* mother, the trial of *Christ*, the crucifixion, and *Christ* being brought down from the cross [50].

## Values of murals

The murals may display an incredibly high level of skill in complex portrayals of various scenes from life. In addition to the aesthetic values, murals have a great history, cultural, religious, educational, tourism, and commercial values and thus are of interest to archaeologists and scientists for research work.

Murals show many historical community events and thus have important historical values. Many murals are termed “peace walls”, “walls that speak”, “walls of expression”, “walls of heritage", “walls of pride”, “walls of empowerment”, etc. [51]. These murals clearly demonstrate information about the culture, lifestyle, production, living conditions, and nature at that time [52]. The music and dance were also clearly shown in the murals [53].

Murals have cultural and religious values because they represent one of the most important facets that embody the identity, traditions, and practices of a country, and they usually reflect the spirit and aspirations of the specific community [54]. For example, the murals can show the alcohol trade, drinking customs, and banquet culture, as well as the types, materials, and decorative patterns of drinking vessels, liquor stores, commerce, and alcoholic beverages [55]. Murals have religious values since there are visual symbols that may reveal underlying meanings and values. In Northern Thailand, some murals illustrate the rebirth of Buddha, and Buddha in heaven being beseeched, Buddha touching the earth, and these murals represent an assertion of certain core values expressed in ancient *Buddhist* symbols [56]. In ancient Greek, where the religion is polytheistic, a large number of murals represent the divine and secular world, i.e., their worship of animals and nature, integration between humans and animals, divinity, and humanity [57].

In addition to the aesthetics, design, and color of murals that can be used as teaching materials. The design of a mural can be used to trigger inspiration, and the color of murals can be used to teach students how to distinguish similar colors [58]. The murals can also play an important role in inclusive education. The murals can only be completed with an emphasis on ideas development and collaborative execution including ideas development, mural design, wall preparation, layout plotting, painting execution, varnishing protection, documentation, appreciation, and reflection [54]. The murals are also representative of the significance and the essence of arts, i.e., passion, imagination, creativity, enthusiasm, endurance, and perseverance [54]. Therefore, incorporating murals into lessons can provide a good opportunity to implement inclusive education.

Mural tourism can stimulate the economy because many murals are among the most visited destinations due to their cultural and natural features. Tourism can be a major contributor to the local economy [59]. Nowadays, the mural landscapes have been regarded as new tourist attractions directly or indirectly both in cities (e.g., *Lisbon* in Portugal, *London* in the UK, *Madrid* in Spain) and rural areas (e.g., *Chemainus* in Canada, *Fanzara* in Spain, *San Gregorio de Polanco* in Uruguay) [60]. Accordingly, the cultural values of murals become an important indicator in increasing an intercultural dialogue based on cultural diversity [61]. It is important to remember that there is an increasing trend in cultural tourism. The murals are highly valuable resources, and it should be borne in mind that sustainable management should be performed to preserve murals [62].

Murals may have great commercial value. Buildings with modern murals often become local landmarks, and they tend to draw more visitors or foot traffic. The property’s market value may be higher with murals [63]. Both the modern and ancient murals can inspire others with a stronger sense of creative flair. There are many traditional elements in the murals, and the design concept, choice of colors, and display part have implications for the current commercial use [64]. Murals and sculptures are the most predominant elements that have been used in advertising [65]. The integration of elements of famous murals into current commercial designs can arouse aesthetic and emotional resonance, expand the audience, and achieve wider dissemination and attention [66]. For example, many elements of the murals in the *Mogao Grottoes* have been widely adopted for commercial use such as advertising and brand design [64, 67].

## Materials composition and research techniques

The commercial pigments for modern murals are largely well-known, while the murals and frescos that were created in the early time were prepared with various mineral powders mixed with lime or water [68]. Currently, most studies pertaining to the materials composition and research techniques for murals are mainly distributed in Europe and Asia which have many traditional murals.

Many types of organic matters were used as binders for the mineral colors such as egg yolk, casein, gelatin, starch, oils from seeds (poppy, flax, and hemp), resins including skin gel from *laccifer lacca*, fish or donkey [69, 70]. The plaster used in the *Goa-ri Tomb* murals in the Republic of Korea was made of oyster shells [71]. The gold powder was also used as pigment for murals in Buddhist caves [72].

Although sample collection allows a precise analysis of materials, non-destructive observation is desired for fragile artworks. There are various methodologies for the on-site characterization of murals. Non-invasive techniques provide chemical information about the materials, and diagnostic methods can reveal structural information and provide multi-layered structures [73]. X-ray fluorescence spectrometer (XRF) [74, 75], macro X-ray fluorescence (MA-XRF) [76, 77], laser-induced breakdown spectroscopy (LIBS) [12, 78, 79], ultraviolet fluorescence [80, 81], infrared [74, 82], microwave imaging [83–85], Laser Raman Analyzer [13, 50, 86], and Terahertz (THz) spectroscope and imaging techniques [84, 87, 88] were widely used to study the materials of the paintings. For example, the Raman analysis can identify the materials of different pigments. Using this method, the composition of the pigments in the fifteenth century *Thubchen Lakhang* monastery was presented, and it was found that the blue pigments were azurite (C_2_H_2_Cu_3_O_8_) and sometimes with lazurite ((Na, Ca)_8_[(S, Cl, SO_4_, OH)_2_|(Al_6_Si_6_O_24_)]), the red and orange paint layers were orpiment (As_2_S_3_) and vermilion (HgS), the brown and green color decorations are composed by red ochre (Fe_2_SO_3_) and malachite (Cu_2_CO_3_(OH)_2_) [89, 90]. For the murals in the rupestrian church *Grotta del Crocifisso* at *Lentini* in Italy, X-ray fluorescence and scanning electron microscopy can easily identify the red, yellow, brown, and green pigments [27]. Using multiple techniques including binocular microscopy, field emission scanning electron microscopy, X-ray spectroscopy, X-ray fluorescence, Fourier transform infrared spectroscopy, thin-film X-ray diffraction, micro-Raman spectroscopy, and Gas chromatography-mass spectrometry, the materials composition in the sixteenth century *Orthodox Syrian Church* in south India were determined. The results showed that the cinnabar (HgS) and malachite (Cu_2_CO_3_(OH)_2_) were mainly used for the red and green pigments, along with minor proportions of lead oxide (PbO), calcite (CaCO_3_), and clay. Plant oils and proteins were used as organic binders [50]. The pyrolysis–gas chromatography/mass spectrometry (Py-GC/MS) with tetramethylammonium hydroxide in double-shot mode can distinguish the various types of binders, detect the marker compounds of organic pigments, and define the nature of alkyd paints [91].

The non-invasive methods also have limitations as diagnostic tools in realistic cases since the materials of murals are numerous and heterogeneous [73]. The Raman, Ultraviolet fluorescence, and infrared can only be used to study the surface layers of the murals because none of them can penetrate thick layers of plaster. X-rays and microwave methods are also limited by dislocations, water damage, and other defects although they can penetrate thick layers. In addition, X-rays and microwaves are suffered from no depth resolution and poor lateral spatial resolution [84]. Optical Coherence Tomography, digital holography, and Terahertz imaging can reveal the structure information and 3D mapping of the multi-layers of murals [73]. The ground penetrating radar can also be used to reveal the different layers of frescos and thus providing valuable information for 3D heritage/historic building information models [92, 93]. Among these techniques, the Terahertz waves can penetrate opaque materials and thus have great potential for the analysis of mural paintings in a non-invasive manner [88]. The Terahertz can obtain fingerprint spectra which are determined by the intermolecular behavior. For example, mercury sulphide (HgS) was widely used as a red pigment and this substance can be recognized by the specific spectra [94].

Although various methods for the determination of the chemical composition and the in-depth delineation of the physical structure of murals have been studied under laboratory experiments, there is a need for incorporating multiple technologies for on-site studies. Due to the similar spectroscopic characteristic and matrix effects of some materials, the accurate identification of chemical compositions of murals is challenging using non-invasive techniques. Recently, the machine learning methods combined with different datasets (e.g., laser-induced breakdown spectroscopy-based dataset, namely, the online RRUFF database which contains an integrated database of Raman spectra, X-ray diffraction, and chemistry data for minerals, https://rruff.info) have been proposed as a useful method to accurately identify the pigments with similar chemical compositions of murals [78, 95]. It is worth noting that there are multiple machine learning methods, e.g., support vector machine, back propagation artificial neural network, K-nearest neighbor algorithm, random forest, and there are also other datasets such as laser-induced breakdown spectroscopy (LIBS) that could be used for the machine learning methods to identify the pigment compositions [78]. The detailed knowledge of chemical composition and physical structure may deepen our understanding of the history of the murals and thus can be helpful to evaluate their authenticities, as well as provide insights into the restoration and conservation of the murals [73]. Further studies including the spectra of pigments, plasters, and other artistic media are required to improve the applicability of this method for mural painting studies.

## Mechanisms of mural contaminants formation and deterioration

As many murals are very old, some of the murals became distorted due to natural conditions such as earthquakes, storms, and rains. Murals often exhibit degradation, shedding, cracking, flaking, and detachment, and there are usually many contaminants on the surface layers of the murals including soot, and microbial colonies. In order to perform effective conservative measures, it is necessary to understand the mechanisms of mural deterioration and the formation of contaminants. Most reports of mural deterioration are conducted in Europe and Asia since these areas occupy a large number of valuable traditional murals.

There are commonly two degradation pathways of mural flaking and detachment, i.e., (1) formation of sulfates depleting and weakening the carbonate layer; (2) solubilization and recrystallization of sulfate salts during the humidity cycles [96]. During this process, moisture is considered one of the most important factors influencing the murals because moisture transformation can lead to salts migration and accumulation, which can further cause efflorescence, flaking, and detachment of the murals [47].

Murals and Frescoes can be susceptible to microbial colonization [97]. Using molecular methods, it has been demonstrated that fungi colonization is the main reason for the dark-spot biodeterioration of the frescos in *Kyiv*, which were created in the eleventh century [98]. The environmental factors including temperature, humidity, exposure to light and Ultraviolet radiation, and the materials used for the arts determine the microbial growth on murals and frescoes [11, 99]. Although the murals and frescoes lacking organic matter are more resistant to microbial biodeterioration fungi and bacteria can affect both organic and inorganic components [98]. The mechanisms underlying the biodeterioration are calcium carbonate glucose agar, casein nutrient agar, pigment secretion, acid and alkali production, and enzymes [100].

Compared with the murals in western churches, murals in China are usually distributed in temples, caves, and tombs. These murals are subjected to long-time contamination by soot due to the combustion of joss sticks, oil lamps, and candles [101]. These processes gradually added organic compounds to the murals, which can support the colonization of bacteria. But later on, abundant fungi can grow on the murals along with the accumulation of organic deposits. Fungi colonizers produce physical and chemical deterioration phenomena [102, 103].

Climate conditions can lead to mural damage. The *Mogao* Caves area is deep in Eurasia inland, far from the sea, and belongs to the arid regions. Currently, the annual precipitation is less than 50 mm, while the evapotranspiration is about 2500 mm [104]. Due to the arid climate, murals and sculptures are largely well preserved, while about 50% of the murals have suffered from deteriorating diseases for several reasons. Many black spots were observed on the murals and cause serious damage to the paintings. These black spots are mainly caused by the fungi communities, and the fungi growth is affected by temperature and relative humidity as well as the history and drawing techniques [11]. The arid climate is also responsible for the frequent sandstorms in this area, and the grottoes are seriously threatened by wind erosion. The higher the height is, the greater wind erosion to the grottoes is [105, 106]. It is worth mentioning that there are some specific risks to the murals according to the location of the murals. For example, rockfall hazards represent a significant risk to the grottoes that are located on cliffs [107].

In addition to the natural conditions, human activities including tourism and inappropriate restoration interventions can also be deteriorative factors for murals. Tourism can disturb the indoor environment although tourism encourages economic growth. High numbers of visitors will contribute to higher humidity and carbon dioxide concentration, which can disrupt the indoor environment and further constitute a risk to the conservation of the murals [108–111]. The airflow in crowed archeological sites contains a high amount of fungal spores including the *Aspergillus* genus, *Malassezia* [109, 112], and these genera are often the dominant genera on the surface of the murals and can lead to biodeterioration of the murals [109, 113]. Without adequate protection measures, airborne fungi are an important source of contamination on the murals, and some murals can be seriously contaminated and even damaged after a large number of visitors come [114]. Inappropriate restoration interventions can directly remove the materials from the murals and may result in secondary damage to the murals [115, 116]. For example, the proteinaceous materials had been incorrectly used for the restoration of a fresco in *Pisa*, and these materials caused serious alterations to the fresco [117].

**Fig. 3:**
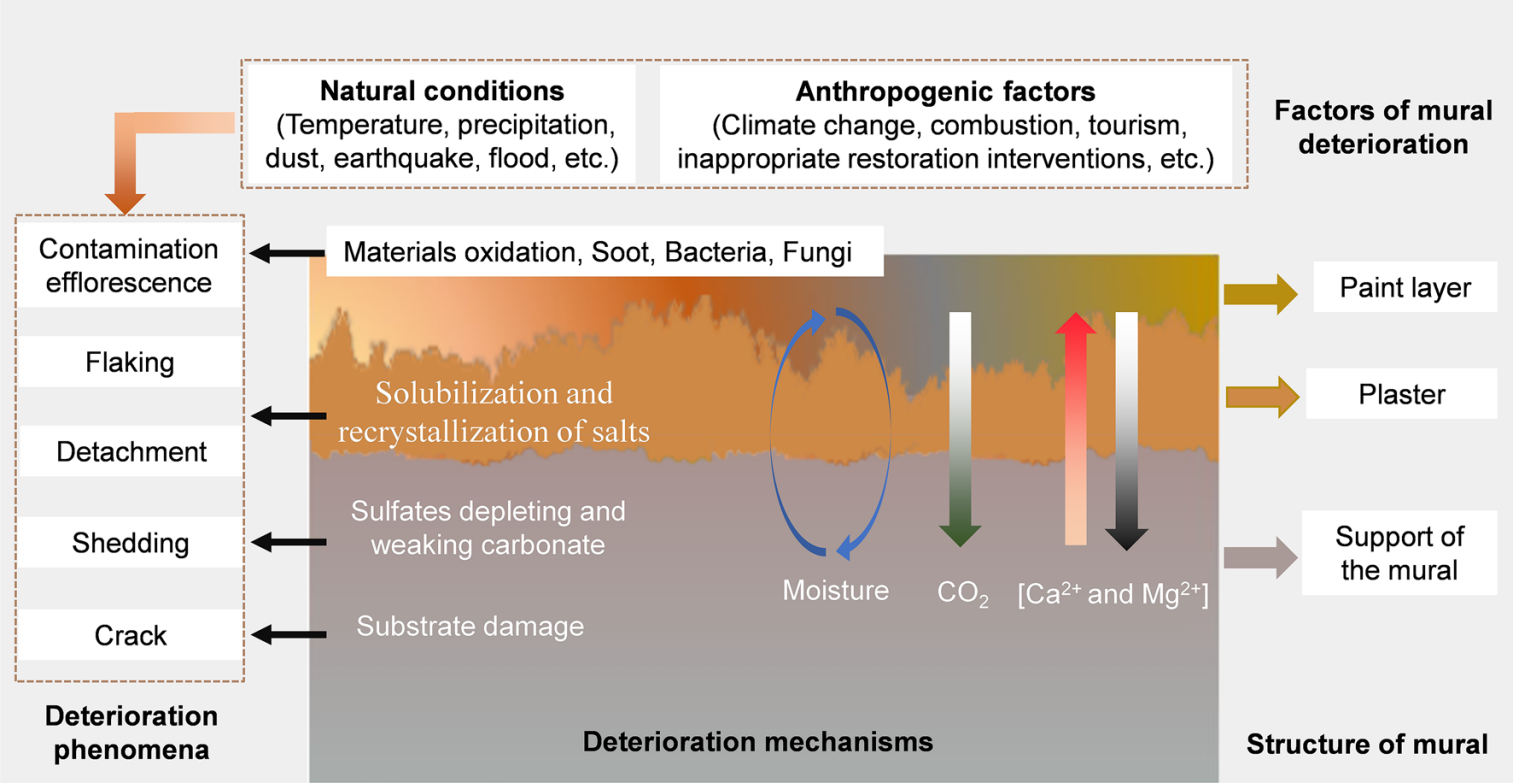
A schematic frame showing the influencing factors and mechanisms of deterioration of murals. The green downward arrow shows the absorption of carbon dioxide, and the pink upward arrow and dark downward arrows show the vertical movement of calcium and magnesium in a mural [118]

Overall, there are many factors that can affect the deterioration of murals via physical, chemical, and biological mechanisms. The main driving factors and mechanisms of murals were summarized (Fig. 3). These factors and mechanisms should be considered in the conservation of murals.

## Measures for mural restoration and protection

The mural is a complex and multilayer system and can be corroded by natural weathering, human activities, and biological effects. Murals face severe damage along with archeological discoveries or deterioration under natural conditions, and the restoration of murals has attracted much attention. The mural restoration includes multiple steps including reinforcement or stabilization by procedures of consolidation of both plaster and pictorial film, repair of the rims of the exposed mortars, surface cleaning, reconversion of the pigment, etc. [119].

Both inorganic and organic materials have been used for mural reinforcement. Inorganic materials such as lime water can bring the aqueous solution into the mural and result in the crystallization of soluble salts [120]. Organic materials, such as acrylic polymers, e.g., Paraloid B-72 (Ethyl methacrylate–methyl acrylate copolymer) and Primal AC33, have been applied to the reinforcement of the murals. Although organic materials have good permeation, adhesion, and transparency, they are suffering from aging problems after thermal oxidation and optical irradiation. The polymer may also block surface pores, which generates mechanical stresses in the mural layer [121]. In recent years, nanomaterials have been used for mural protection [122], and the nanocomposite PAAG@Ca(OH)_2_ displayed a promising application for the reinforcement of murals due to the high porosity, strong adsorption, appropriate hydrophilicity, good permeability, and strong adhesion to mural pigments [15].

Many processes lead to abundant contaminants appearing on the mural surfaces. The contaminants affect the aesthetic values of murals and accelerate the deterioration of the murals. In order to reveal hidden details or information and prolong the life of murals, cleaning of surface contaminants is critical for murals conservation, while removal of surface deteriorated overlayers from murals is a delicate and complicated work due to the multitude of materials within the murals and the high sensitivity of materials to environmental factors such as heat and light. Traditional methods such as chemical solvent and mechanical cleaning can remove contaminants. However, these methods can also damage the mural surface [123]. Some nanomaterials are capable of holding large amounts of liquid, but have a low liquid penetration rate into the substrate. Both model and in situ tests showed that using some nanomaterials can significantly reduce the ingrained dirt on murals when compared with traditional methods [124]. Recently, lasers have been widely used to clean cultural heritage and protect features and this method can effectively remove dust and soot on mural surfaces. However, laser cleaning can lead to damage to the pigment layer due to heat mechanisms. In addition, more damage is likely to occur under ultraviolet laser irradiation than near-infrared laser irradiation because the former has a higher photon energy of electrons [125, 126]. Ultraviolet laser irradiation is effective to remove contaminants on stone substrates [123, 127, 128]. Obviously, although laser cleaning is a promising approach to process unwanted surface layers on murals, the physiochemical properties of the material, laser fluences, and the number of pulses should be carefully assessed before the implementation of contamination removal. In addition, in situ monitoring of the cleaning process is also necessary to safeguard the original painting [123, 125].

Microorganisms are causative agents of biodeterioration in murals, while they can be positively used for the cleaning of murals. The bio-cleaning method, which uses the exoenzymes produced by selected microbial strains, can remove a widely utilized consolidant, adhesives, nitrate, and sulfate salt, and oil from the painted surfaces [129]. The bio-cleaning methods are non-toxic, non-aggressive, non-invasive, and highly specific for removing surface contaminants, and thus represent a promising result in the field of bio-cleaning of murals [130]. It should be borne in mind that some of the exoenzymes could not be completely removed during the restorations and these compounds may cause aesthetic damage to the murals over time [131]. Currently, the exoenzymes that could be potentially used for mural cleaning are not produced at an industrial, and they are not commercially available. Consequently, the production cost of enzymes represents a major drawback for the widespread use of bio-cleaning. Meanwhile, more studies are required to systematically assess the efficiency of these methods as well as the potential effects of remaining exoenzymes of the murals [129]. In addition to the bio-cleaning methods to remove the surface contaminants, the X-rays can be potentially used to cure biodegradation by inhibiting the growth of pathogens on murals in a green way [132].

The lead-based pigments, particularly red lead (Pb_3_O_4_) and lead white (2PbCO_3_·Pb(OH)_2_) were among the most used materials for pigments in murals. These materials tend to be blacked with time due to the oxidation to plattnerite (PbO_2_). The proposal of scraping away the darkened part and repainting is not acceptable because it would destruct many original materials [133]. Chemical reagents can be used for the reconversion of these pigments, but these methods may put pictorial layers at risk [134]. Continuous wave laser heating can be used for the reconversion of minium (Pb_3_O_4_) and massicot (PbO) in murals, while the formation of massicot as an intermediate product is a promising reconversion of lead white [13]. The distortions can lead to mistakes in mural identification and classification, while traditional restoration of damaged artworks requires talented artisans, which are difficult to find these days. Therefore, virtual restoration of the murals may have great potential for mural conservation and research [17, 135]. Using computer technology, digitally repaired damaged murals can be helpful for the virtual display of the murals, and it has been shown that cloud edge computing could be a useful tool to automatically identify and repair the cracks of murals [136]. Deep learning and visual reality technology can build a digital recognition model which can accurately identify the colors and drawing materials of each specific color and thus be of great significance for the preservation simulation of murals [137, 138]. However, deep learning methods usually require a large amount of data and computational resources. Therefore, the establishment of some feasible methods for conservation and the digital documentation process is still needed to support heritage institutions [139].

It should be stressed that a good understanding of the material compositions, structures of the murals, formation of contaminants, and other deterioration mechanisms are needed before the restoration process [26–28]. Understanding the deterioration mechanisms can provide a scientific basis for mural conservation and remedial treatments. For example, when microbial communities are detected, measures such as frequent cleaning, adequate ventilation, and indoor climate management can be carried out to prevent mural deterioration [100]. On the contrary, the restoration of murals with an insufficient understanding of the materials and mechanisms of the deterioration can lead to considerable damage. In the 1980s, China restored many murals by applying epoxy adhesives, and these materials showed shrinkage and gradual deformation caused by fluctuating environmental conditions. Nowadays, it is difficult to dismantle these structures and safely remove the support layer made of glass fiber bonded with epoxy adhesives [140].

## Emerging opportunities and challenges for mural conservation

Since the first industrial revolution, our science and technology developed rapidly, which further propelled the economy forward at the global scale [141]. Meanwhile, the increasing human activities, especially the burning of fossil fuels and changing land use types, lead to rapid climate change such as increasing temperature, precipitation, and extreme events [142]. These changes bring both opportunities and challenges for mural conservation.

New technologies provide more useful tools to study the murals’ chemical composition, physical structures, and deterioration mechanisms. The traditional single-view scene cannot show all deterioration forms of murals because changes may happen in materials and plaster layers. The depth, hyper-spectral, and multi-view cameras can be used to capture the 3D structure of deterioration [143]. It has been proposed that a multi-path convolutional neural network, which takes multiple lighted image patches as inputs and outputs the corresponding label of the center pixel, can rapidly detect mural deterioration [144]. Recently, machine learning methods have been widely for murals study, which can improve the accuracy of composition detection [16, 17]. In addition, based on pigments and physical strata of the murals from multi-band imaging techniques, it is possible to virtually reconstruct the original color and appearance of the faded murals. These technologies have been successfully used in some murals in the *Mogao Grottoes* in China [145]. The application of molecular and microbial technology provides deep insights into the processes and mechanisms of microbial deterioration of murals [146, 147].

Murals are tourist resources, and tourism development can boost the local economy. Tourism can also raise public awareness of the values of murals and benefit the education of aesthetics, history, culture, etc. [60, 148]. Tourism is expected to increase in the future despite the shrinkage of the tourist market in recent years due to COVID-19 [149, 150]. However, visitor walking can increase the bioaerosols particles. Although the installation of glass barriers near the wall can decrease the deposition number of bioaerosols particles and thus can protect murals from the deposition of bioaerosols particles, visitors can still change the moisture and air flow, thus leading to further deterioration of murals [114]. It has been well recognized that it is important to preserve the cultural landscape of mural destinations, and strict measures should be performed. In many areas, there is a lack of cooperation between the key stakeholders in policy formulation and implementation, which could pose a threat to the balance of mural protection and sustainable development of tourism [108].

Climate change threatens mural conservation. Many murals were built before the industrial revolution, a period in which the climate was very different from what it is today. Currently, there are increasing trends in air temperature and air pollution. The precipitation also shows an increasing trend with great annual fluctuations. These changes likely induce chemical and biological deterioration of murals. Consequently, studies revealing the deterioration of murals with climate change are needed, and it is necessary to predict what would happen in the murals, and what methods can be applied for the conservation of the murals under a changing climate [151].

**Fig. 4:**
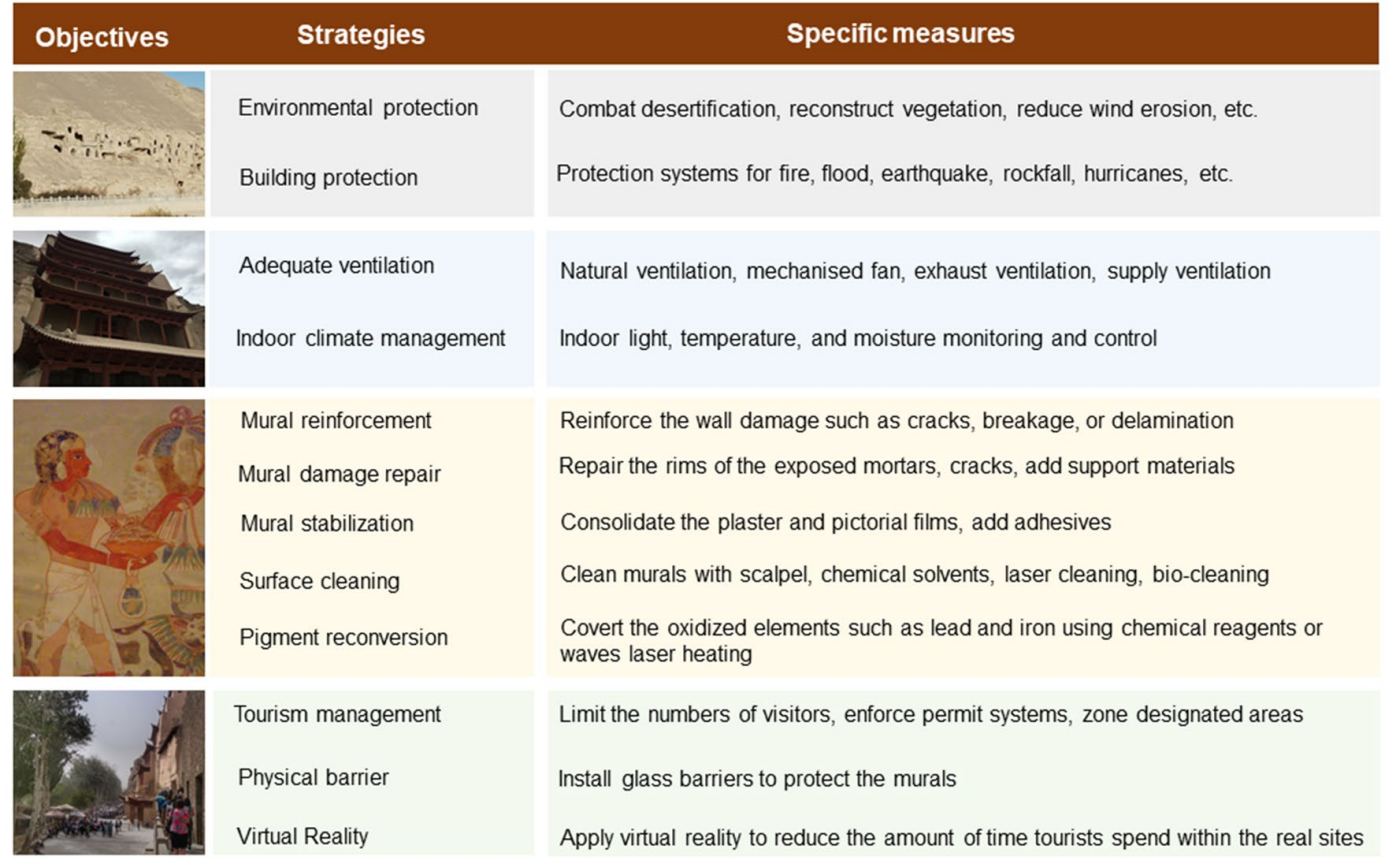
Some strategies for restoration and protection of murals. The upper two photos were shot by the authors. The Egyptian mural is credited to “Ancient Egyptian Murals at the Metropolitan Museum of Art in New York (3)" by mharrsch, and the image is licensed under CC BY 2.0. The bottom picture is credited to “Mogao, by txikita69”, and this image is licensed under CC BY-NC-SA 2.0

Based on the above descriptions, the main strategies and specific measures for the conversation of murals were summarized (Fig. 4). In short, preservation, restoration, and utilization of murals should be considered in a broad context.

## Conclusions and remarks

Murals are distributed widely around the world. The most studied murals are distributed in Mexico, Ireland, Spain, China, Italy, and the USA. The murals in Mexico and Ireland are mainly modern murals. In Europe, many murals are associated with buildings such as churches that were built in the medieval period. The most famous murals in China are associated with grottoes and have a long history that can date back to the third century. The murals have multiple values including aesthetics, history, cultural, educational, tourist, and commercial values. Many methods including chemical analysis, non-invasive spectroscopic methods, Terahertz, and ground penetrating radar have been used to detect the chemical composition and physical structures of the murals. The most common phenomena are degradation, shedding, cracking, flaking, and detachment. Mural deterioration can be induced by both natural of anthropogenic factors. The application of microbial and molecular methods has deepened our understanding of the mechanisms of microbial deterioration in murals. Stabilization, repair, surface cleaning, and pigment reconversion are the common measures of mural restoration. The rapid development of computer science provides new tools including machine learning algorithms to study and conserve murals. The major achievements of mural study and protection during the past decades include the materials composition using new techniques, mechanisms of mural deterioration, computer science and technology used for mural restoration and protection.

Although many progresses have been achieved in the study and conservation of murals in the past decades, our knowledge is far from sufficient to understand the deterioration of murals including color-changed pigments and fading of the image lines, and these knowledge gaps hinder our ability to reconstruct the murals in detailed information. Therefore, more studies are required to understand the composition, structure, and deterioration mechanism of the murals. New technologies such as bio-cleaning, and nanomaterials are potentially useful for the conservation of murals. The major challenges for mural study and conservation are the rapid climate change and the balance of mural protection and sustainable development of tourism. Therefore, in order to achieve sustainable development of murals, tourism management, and climate change should be also considered in the study and the conservation of murals in the future.

## Data Availability

Not applicable.
